# Hippocampal CA1 Transcriptional Profile of Sleep Deprivation: Relation to Aging and Stress

**DOI:** 10.1371/journal.pone.0040128

**Published:** 2012-07-05

**Authors:** Nada M. Porter, Julia H. Bohannon, Meredith Curran-Rauhut, Heather M. Buechel, Amy L. S. Dowling, Lawrence D. Brewer, Jelena Popovic, Veronique Thibault, Susan D. Kraner, Kuey Chu Chen, Eric M. Blalock

**Affiliations:** 1 Department of Molecular and Biomedical Pharmacology, University of Kentucky College of Medicine, Lexington, Kentucky, United States of America; 2 Department of Microbiology and Immunology, University of Texas Medical Branch, Galveston, Texas, United States of America; 3 Department of Biology, Dickinson College, Carlisle, Pennsylvania, United States of America; 4 Sanders-Brown Center on Aging, University of Kentucky, Lexington, Kentucky, United States of America; 5 Department of Neuroscience Cell Biology Physiology, Wright State University, Dayton, Ohio, United States of America; Tokai University, Japan

## Abstract

**Background:**

Many aging changes seem similar to those elicited by sleep-deprivation and psychosocial stress. Further, sleep architecture changes with age suggest an age-related loss of sleep. Here, we hypothesized that sleep deprivation in young subjects would elicit both stress and aging-like transcriptional responses.

**Methodology/Principal Findings:**

F344 rats were divided into control and sleep deprivation groups. Body weight, adrenal weight, corticosterone level and hippocampal CA1 transcriptional profiles were measured. A second group of animals was exposed to novel environment stress (NES), and their hippocampal transcriptional profiles measured. A third cohort exposed to control or SD was used to validate transcriptional results with Western blots. Microarray results were statistically contrasted with prior transcriptional studies. Microarray results pointed to sleep pressure signaling and macromolecular synthesis disruptions in the hippocampal CA1 region. Animals exposed to NES recapitulated nearly one third of the SD transcriptional profile. However, the SD -aging relationship was more complex. Compared to aging, SD profiles influenced a significant subset of genes. mRNA associated with neurogenesis and energy pathways showed agreement between aging and SD, while immune, glial, and macromolecular synthesis pathways showed SD profiles that opposed those seen in aging.

**Conclusions/Significance:**

We conclude that although NES and SD exert similar transcriptional changes, selective presynaptic release machinery and Homer1 expression changes are seen in SD. Among other changes, the marked decrease in Homer1 expression with age may represent an important divergence between young and aged brain response to SD. Based on this, it seems reasonable to conclude that therapeutic strategies designed to promote sleep in young subjects may have off-target effects in the aged. Finally, this work identifies presynaptic vesicular release and intercellular adhesion molecular signatures as novel therapeutic targets to counter effects of SD in young subjects.

## Introduction

Age-related cognitive deficits are highly prevalent and constitute an important health risk in the human population (reviewed in [1]). They can presage development of neurodegenerative disease [2], [3], [4], and are a primary reason for elderly placement in assisted living facilities [5]. Perturbations in sleep are also a common complaint among the elderly and include circadian advance, sleep fragmentation, and insomnia [6], [7], [8], [9], [10], [11]. Healthy young adults show some aging-like phenotypes when deprived of sleep, including daytime sleepiness [12], metabolic syndrome-like changes, and cognitive deficits [13], [14], [15]. This is consistent with work suggesting that sleep promotes memory [16], [17], [18], [19], possibly through slow wave influence on synapses [20], [21], [22] and/or promotion of macromolecule synthesis [23]. Further, numerous studies have pointed to the deleterious effects of stress and stress hormones on brain function and a major hypothesis of aging (the glucocorticoid hypothesis) posits that continued exposure to stress and stress hormones over age is a fundamental cause of age-related deficits in various systems (reviewed in [24], [25], [26]). Thus, dysregulated sleep and stress seen with age might contribute to age-related functional changes. Despite the seemingly similar effects of age, stress and sleep deprivation (SD), and the high prevalence of sleep changes and new onset stress with age, relatively few studies have tested for a molecular relationship between the influences of SD, stress, and aging on brain tissue.

Here, we hypothesized that an aged or stressed animal’s hippocampal transcriptional profile would be similar to that of a sleep-deprived subject. We tested a prediction of this hypothesis by sleep depriving young animals and statistically testing for aging- or stress-like transcriptional phenotypes in the hippocampus. Young F344 rats were sleep deprived for 24 or 72 hours using the modified multi-platform ‘flower pot’ method [27], [28]. Blood corticosterone levels, adrenal weights, body weights, and hippocampal CA1 gene expression profiles were measured. A second set of rats was exposed to novel environment stress for 24 or 72 hours to help control for non-specific stress effects of the environmental change necessitated by the sleep deprivation protocol. In a third set of animals, the SD protocol was applied and gene products were validated at the protein level using Western blots. Data were subjected to bioinformatic analysis and contrasted with results from prior transcriptional profiling studies as noted in Results.

Transcriptional comparison suggests SD, stress and aging interact with a similar subset of genes within the hippocampal transcriptome. However, although there was strong directional agreement between SD and NES, notable disagreements between SD and aging were seen, including opposite inflammatory and glial expression changes. Our studies also identified SD-specific genes and gene profiles that may represent targets for therapeutic intervention. These include the previously identified *Homer1* as a potential sleep regulation molecule, but also other novel candidate genes involved in pathways related to synaptic function, vesicular release and intercellular adhesion. However, because of the more complex relationship between SD and aging, it is also likely that sleep or stress-related interventions designed for younger subjects may have off-target effects in aged subjects.

## Materials and Methods

### Ethics

Use of vertebrate animals was carried out in strict accordance with the recommendations in the Guide for the Care and Use of Laboratory Animals of the National Institutes of Health. The protocol (# 2008-0347) was approved by the University of Kentucky Office of Research Integrity Institutional Animal Care and Use Committee. Euthanatising (CO2) anesthesia was used and all efforts were made to minimize suffering.

### Subjects

Male Fischer 344 rats (3–5 months old, Harlan, Indianapolis) were housed 2 per cage and maintained on a 12∶12 light/dark cycle in the housing facility with *ad libitum* food and water. Subjects were acclimated to the housing facility for two weeks prior to initiating the study. All procedures involving animals adhered to the NIH Guide for the Care and Use of Laboratory Animals and were approved by the Animal Care and Use Committee at the University of Kentucky. 110 animals were used, 38 in cohort 1 (one animal was not included because of poor health), 32 in cohort 2, and 40 in cohort 3. Subjects in cohorts 1 and 2 were used for microarray analysis and were assigned to one of 5 treatment groups: Home Cage (HC) n  = 17; 24 hour sleep deprivation (24SD) n  = 16; 72 hour sleep deprivation (72SD) n  = 20; 24 hour novel environment stress (24NES) n  = 10; 72 hour novel environment stress (72NES) n  = 6. SD and HC animals in cohort 1 were trained on the one-way active avoidance task prior to SD. No effect of training was noted in stress or microarray measures (not shown). Cohorts 1 and 2 were combined for microarray analyses. Animals in the third cohort were used for protein measures and included HC n  = 16; 24SD n  = 12; and 72SD n  = 12.

### Sleep Deprivation

The multiple platform modification [29] of the classic ‘flower pot’ [28] technique in which animals are placed on an elevated platform over water was used. Based on prior research regarding the stress-reducing action of pairing socially familiar animals [27], long-standing cage mates were housed together in sleep deprivation chambers. Pairs of SD animals (cage mates) were p laced in large 3′x2′x2′ water tight polycarbonate chambers covered with stainless steel grating (Braintree Scientific, Braintree, MA). The chamber was filled with water to a depth of 2 inches. Twenty four inverted clay flower pots served as elevated platforms (4 inch diameter) within each chamber, allowing the animals to stay dry, move around, and access food and water supplied through the grated chamber lid.

### Novel Environment Stress (NES)

Procedures for reducing/preventing sleep are often stressful, as those for inducing stress can be sleep-depriving [30]. To help address this, for a subset of animals, the sleep depriving component was prevented by placing a rubberized metal grid over the platforms. Thus, NES animals were exposed to the same novel environment as the SD animals, but were not constrained from sleeping. These NES animals paralleled the sleep deprivation exposure duration (24 hour- n  = 10, and 72 hour- n  = 6) and hippocampal microarray signatures also were collected.

### Tissue Isolation

On the day that animals were removed from the SD chambers, they were deeply anesthetized with CO_2_ gas and decapitated. Trunk blood was collected, immediately centrifuged to isolate serum, and aliquots were frozen (-80 C) until further use. Corticosterone (CORT) radioimmunoassay (RIA) was used to measure CORT levels (RIA kit, ICN Biomedicals, Costa Mesa, CA) according to manufacturer’s directions. Briefly, 10 ul of serum was diluted 1∶200 in αβαγ buffer and incubated in antibody for 2 hours at room temperature. All samples were run in duplicate and averaged.

Brains were removed and immediately placed in oxygenated artificial cerebrospinal fluid (aCSF) containing (in mM) 114NaCl, 2.5 KCl, 2MgCl_2_, 30 NaHCO_3_, 10 Glucose and 2.2CaCl_2_. Hippocampi were dissected as described previously [31], [32]. Briefly, hippocampi immersed in 0°C oxygenated aCSF were placed on a chilled glass petri dish, and the CA1 region dissected away from dentate gyrus, CA3 and entorhinal cortex along the long axis of the hippocampus. CA1 tissue was flash frozen and stored at -80°C until further use.

### RNA Extraction

RNA was extracted from each CA1 tissue block, as described previously [31], [32], [33] using TRIzol (Invitrogen) followed by ethanol precipitation, and was reconstituted in RNase-free water. For each sample, 20 µg of biotin-labeled cRNA was generated from 5 µg of total RNA according to manufacturer’s instructions (Affymetrix). 20 µg biotin-labeled cRNA was applied to a rat RGU34A GeneChip (Affymetrix) for hybridization (one chip per animal).

### Microarray Protocol

The Affymetrix Gene Chip Operating Software (GCOS) quality control algorithm showed no significant difference (p>0.2, 1-ANOVA) for three quality control measures: scaling factor (2.45+/−0.06, target intensity of 500); GAPDH 3′:5′ (1.04±0.01); and β actin 3′:5′ (1.31±0.02). There was no significant difference among treatment groups regarding percent of probe sets rated present (39.5±0.03% present); p>0.4, 1-ANOVA). However, GCOS measures of background signal (74.9±2.1 cohort 1; 59.8±2.3 cohort 2) and RawQ (2.3±0.1 cohort 1; 1.9±0.1 cohort 2), as well as a chip x chip correlation matrix of signal intensity data (not shown), pointed to a global decrease in background signal intensity in Cohort 2. To control for this, each gene’s expression values for HC, 24SD and 72SD was standardized separately within cohort 1 and cohort 2, and then the two cohorts were combined. This retained the variability of each cohort, but removed the ‘cohort effect’ from the data. The raw signal intensity values (MAS5) and scanned images (.cel files) have been deposited to the MIAME compliant Gene Expression Omnibus (GEO) database ([34] - accession #GSE34424).

Values were transferred to Excel (2007, Microsoft), Bioconductor [35], or MultiExperiment Viewer (MEV, [36]), and integrated with annotation data (Affymetrix, June 2011). Pre-statistical filtering retained ‘A’ grade probe sets that were uniquely annotated with gene symbols and had at least 6 presence calls across all 69 HC and SD chips. This criterion was selected based on factorial analysis demonstrating <5% probability of 6 or more chips being rated present for a given probe set by chance (Blalock, unpublished observations).

### Biological Pathway Identification

Functional processes significantly overrepresented in the data set were determined using the Database for Annotation, Visualization and Integrated Discovery (DAVID) overrepresentation tool [37] on the Gene Ontology (GO) database [38]. The ‘table clustering’ option and streamlined ‘GO-FAT’ subset of the GO were used for analysis. A single illustrative process from each cluster (p<0.05; kappa statistic/‘EASE score’) [39], populated by between 3 and 50 genes, is reported in Results.

### Overlap Analysis

For comparisons across different transcriptional profiles (e.g, sleep deprivation vs. aging, or sleep deprivation vs. novel environment stress), statistical similarity is determined by ‘overlap analysis’ as previously reported [40], [41], [42], [43], [44], [45]. Briefly, a total number of genes available for testing across two studies (common background) is established based on gene symbol level annotation and high quality signal in both studies. Among this set of background, the number genes significant in both studies (the ‘overlap’) is statistically contrasted with the number of genes one would expect in the overlap by chance using the binomial test [46].

### Western Blot Experiments

A separate cohort of animals was exposed to the SD paradigm (n  = 12–16/group; HC, 24SD and 72SD), and their hippocampi removed for protein extraction and Western blot analysis. Briefly, intact hippocampi were flash-frozen in liquid nitrogen and stored at -80°C until use. Hippocampi were homogenized in 500 µl 0.3 M sucrose buffer containing protease inhibitors (300 mM sucrose, 75 mM NaCl, 10 mM Tris pH 7.4, 20 mM EDTA, 20 mM EGTA, 1 µg/ml pestatin A, 10 µg/ml leupeptin, 20 µg/ml, 200 nM PMSF, 8 µg/ml calpain inhibitor I & II). Tissue was centrifuged in a microfuge (3500 rpm/1000 g×5 min) to remove cellular debris. Supernatant (membrane/cytosol fraction) was collected and stored at -80°C until further use.

Protein concentration was determined by the Lowry method with no significant difference among treatment groups (35.2±1.2 µg/µl; p  = 0.76; 1-way ANOVA). For each sample, 200 µg of protein was loaded on Bio-Rad gradient gels (4–20%). Proteins were resolved by electrophoresis and transferred to nitrocellulose membranes for quantitative Western blot as described previously [47]. Membranes were incubated at 4°C overnight in I-Bloc (Tropix) along with primary antibodies, which included: Agrin (1∶000 dilution, Chemicon catalogue MAB5204, monoclonal); Adrb2 (1∶5000, Chemicon catalogue AB5617, polyclonal); Sgk-1 (1∶1000, Upstate catalogue # 07-315, polyclonal); Nr3b (1∶2500, Upstate catalogue # 07-351, polyclonal), Neurexin 1 (1∶1000, Biovision catalogue # 3428-100, polyclonal); EAAC1 (1∶500, Upstate catalogue # MAB1587, monoclonal); and GluR2/3 (1∶5000, Upstate catalogue # 07-5998, polyclonal). Primary antibodies were detected using the appropriate HRP-conjugated secondary antibody, diluted in I-Bloc at 1∶10,000, and detected using the ECL-plus Western kit (GE Healthcare). Protein levels were quantified using a Storm 860 PhosphoImager (MolecularProbes).

### Other Microarray Studies

Published microarray studies of aging rat hippocampus, along with their Gene Expression Omnibus data deposition references include: GSE854 [31]; GSE5666 [33]; GSE9990 [32]. Procedures for statistical comparison of the present work to these published studies are similar to those published previously [40], [45], [48] except as noted in Results.

### Statistical Analysis

All statistical analyses were performed using SigmaStat, SAS, and/or custom formulae in Excel (Microsoft, 2007). Outlier values (>2SD of treatment mean) were treated as missing values. For microarray data, the error of multiple testing for genes rated significant (1-ANOVA, p≤0.05) was estimated by the False Discovery Rate (FDR) procedure [49], with median FDR reported. Significant genes were further investigated with a post-hoc template matching strategy [32], [45].

### Interpretation Caveats

The following are important considerations/limitations regarding the interpretation of this data. First, mRNA and protein levels are used to infer functional changes, but are not direct measures of function. Second, gene products are pleiotropic and can play different roles depending on the cell/tissue type and temporal context of their expression. Third, although enriched in neurons, the CA1 region of the hippocampus is not exclusively neuronal. Astrocytic, oligodendrocytic (richly myelinated perforant path and alveus), microglial and vascular cells/tissue are transcriptional contributors. Fourth, the SD profile can differ across brain regions [50] and, probably, species. To help guard against mis- (or over-) interpretation, particular attention is paid to genes that participate in larger, statistically significant functional groups, are known to be expressed in brain tissue, and/or have been found to change in prior studies examining similar phenomena in similar tissue.

## Results

### Stress-related Measures

**Figure 1:**
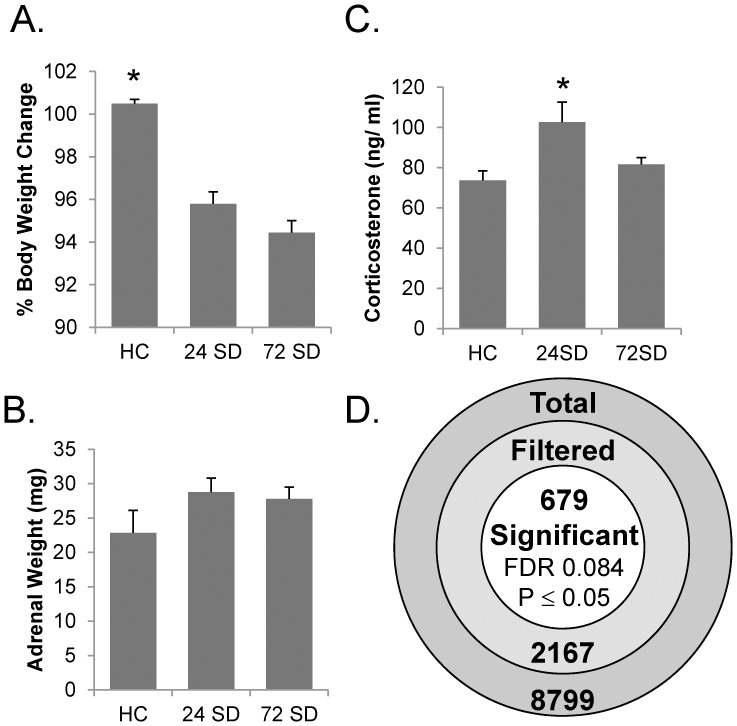
Sleep deprivation reduces body weight, increases corticosterone, and causes detectable changes in hippocampal gene expression. A. Body weight was significantly reduced in all SD animals from both cohorts. Weights measured at the end of the study are expressed as a percentage of the body weight measured at the beginning of the study, a 4 day span (1-way ANOVA [* F_2, 49_ = 29.1, p  = 4.8×10^−9^]). B. Adrenal weights measured from animals in cohort 1(HC n  = 9; 24SD n  = 9; 72SD n  = 10) were not significantly increased (p  = 0.22). C. Corticosterone levels from animals in cohort 2 (HC n  = 12; 24SD n  = 9; 72SD n  = 13) were significantly increased at 24SD (1-way ANOVA [F_2,31_ = 5.93, p  = 0.0066]; *post-hoc Tukeys). D. Of 8799 total probe sets, 2167 were rated present and had unique gene symbol level annotations. These were tested by 1-way ANOVA across HC, 24SD, and 72SD groups. A total of 679 genes were rated significant (p≤0.05). The False discovery rate (FDR) procedure estimates that 8.4% of these results are significant due to the error of multiple testing.

We measured three stress-related outcome variables: body weight, adrenal size, and corticosterone level in control and SD animals. Although not a large effect (∼5%), there was a consistent and significant weight loss associated with sleep deprivation (Fig. 1A). Adrenal weights were not significantly increased in SD animals (Fig. 1B). CORT levels (Fig. 1C) were measured at the circadian nadir. 24SD but not 72SD subjects showed a significant elevation. Control CORT levels are in range with those reported in other studies [51], [52].

### Gene expression Patterns

The CA1 hippocampal subregion (Methods) was used for gene expression microarray analysis. Data were filtered (Fig. 1D) to remove probe sets that were poorly annotated, redundant, or had weak expression levels. One-way ANOVA identified nearly 700 genes significantly influenced by SD. Approximately 8.4% of those are likely to be present due to the error of multiple testing as estimated by the False Discovery Rate (FDR) procedure. We consider this a fairly strong result, particularly compared to studies on normal aging, where FDRs are typically in the 20-25% range. However, the present work also has a higher n per treatment group, likely increasing statistical power.

**Figure 2:**
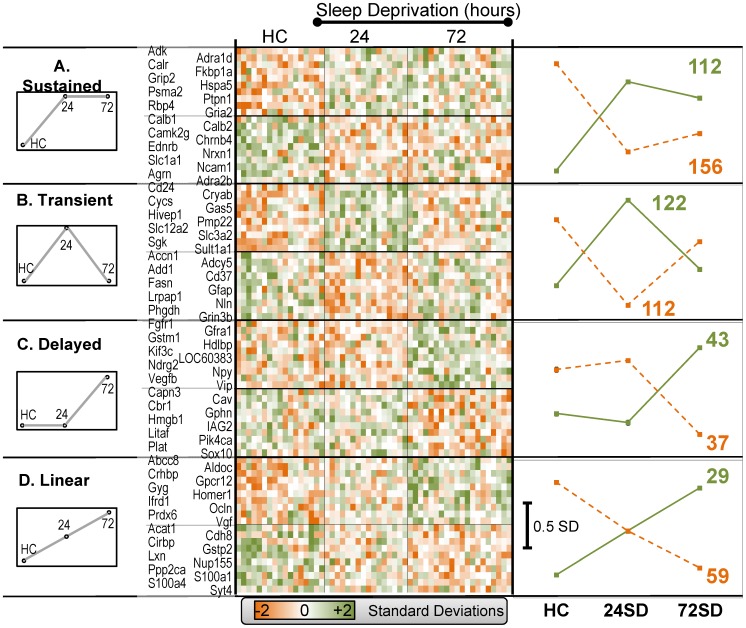
Expression patterns for significant genes are shown. **Left:** Artificially constructed templates (**A. Sustained; B. Transient; C. Delayed, and D. Linear)** were used to partition genes into specified patterns. The treatment group mean expression value for each significant gene was correlated with each of the four templates and the gene was assigned to the template with the highest |R|. Positive correlations are considered ‘increased’ with SD, negative correlations are considered ‘decreased’. **Center:** Heatmap for 10 representative genes assigned to each template and direction are shown. Data are expressed in standardized units and color coded (lower, color scale) by standard deviations from the mean. **Right:** Averaged results for all genes in each template are graphed (positive  =  solid green; negative  =  dashed orange; # genes in each pattern reported). Note: error bars plotted but obscured by symbols.

The combination of significance and direction of change for significant genes constitutes the gene expression pattern for a single gene. Here, we used template matching [32] to parse ANOVA-significant genes into expression patterns. Four experimenter-defined expression patterns (templates) were constructed to examine SD effects: sustained, transient, delayed, and linear (Fig. 2 A, B, C, D). Treatment group mean gene expression for every significant gene was correlated with each of these four templates, and each gene was assigned to the template with which it most strongly correlated. Positive correlations were interpreted as “increased” with sleep deprivation, and negative correlations as “decreased”. The complete list of all significant genes, along with their template assignments, mean expression values and 1-way ANOVA p-values, are provided in Table S1.

Heat maps (Fig. 2, center) show color-coded standardized expression values for 10 representative genes from each pattern for each subject. On the right, the averaged expression values for all significant genes assigned to each template are shown, along with the total number of genes assigned to that template. Interestingly, most significant genes were assigned to either the sustained or transient templates. Further, the 72SD group tended to show a lesser (albeit non-significant) tendency to recover back to HC values.

To determine whether the number of genes categorized into each expression pattern was significant, we ran a Monte Carlo simulation. For the simulation, the data table used the same number of chips (columns), and same number of genes (rows), and the same ANOVA and post-hoc template matching strategy as the original microarray data set. However, all signal intensity values were replaced with randomly generated numbers. Then the number of significant “genes” that were categorized in each template was counted. This process was repeated 1000 times, with new randomly generated numbers each time. Out of these 1000 iterations, on average, the random data identified an average of 14 ‘genes’ in the sustained and delayed templates, 19 ‘genes’ in the transient template, and 9 ‘genes’ in the linear template. These results indicate that different templates have different random probabilities of having genes assigned to them, and this should be taken into account during this type of analysis. However, the actual data for all templates (Fig. 2, right) far exceed these Monte Carlo estimated values (p≤1.3×10^−8^ for all templates, binomial test). Thus, all template-identified genes are occurring at a significantly greater frequency than expected by chance.

### Protein Measurements

**Figure 3:**
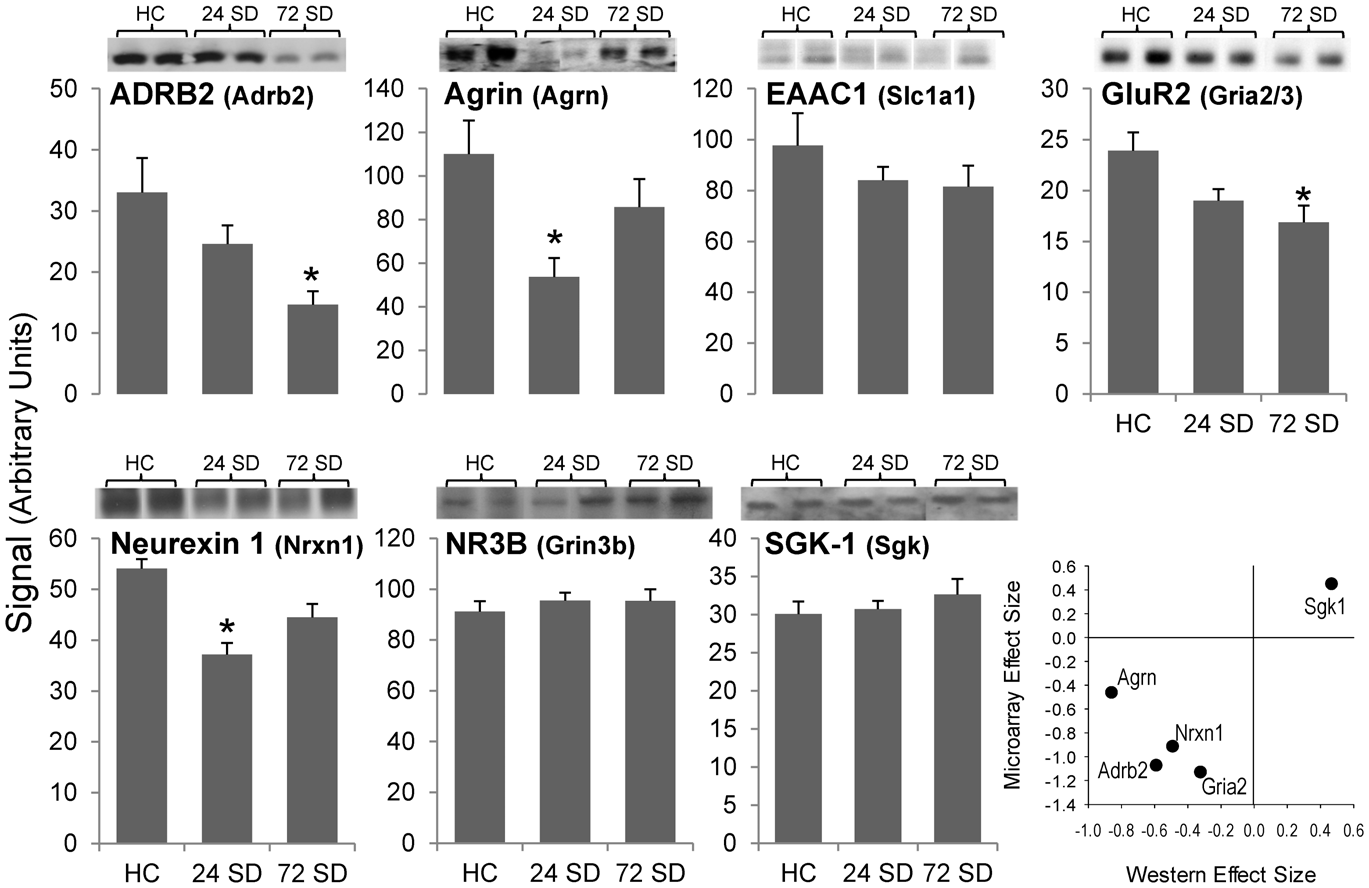
Based on results from microarray analysis, the protein products of 7 genes were analyzed. Western blot analysis was performed on a separate cohort of sleep deprived and control subjects (n  = 12–16/group; HC, 24SD, and 72 SD). Adrenergic beta 2 receptor (**Adrb2**), agrin (**Agrn**), AMPA-selective glutamate receptor 2 (**GluR2**/*Gria2*), and neurexin 1 (**Nrxn1**) were significantly downregulated at both the protein and mRNA levels. Excitatory amino acid transporter (**EAAC1**/*Slc1a1*) and serum glucocorticoid kinase 1 (*Sgk*/**Sgk-1**) were not significant at the protein level. Representative immunoblots from Western analysis are shown for each gene product. **Last Panel:** Plot of effect sizes (differences in mean expression expressed in standard deviations) for selected mRNA (microarray) and protein (Western) results for individual gene products shows general agreement in direction and significance of change.

Microarray results informed our decisions regarding selection of gene products for measurement at the protein level. A separate cohort of animals was subjected to the SD protocol and hippocampi were removed for Western blot analysis on 7 proteins: ADRB2, Agrin, EAAC1, GluR2, Neurexin 1, NR3B, and SGK-1 (Fig 3). These proteins were chosen because our initial analysis of microarray data pointed to these neuronal function downregulation or stress response upregulation with SD. Western blot analysis showed directional agreement for 5/7 products, 4 of which were significant (Adrb2*, Agrin*, GluR2*, Neurexin 1*, Sgk-1; * significant). With the exception of Sgk-1, protein analysis was focused on neuronal products and appeared to agree with generalized downregulation seen at the mRNA level. We interpret this as fairly strong agreement especially considering that the experiment was done in a separate cohort of animals.

### SD Gene List Consolidation

To streamline comparison with other gene expression data, each ANOVA-significant SD gene’s expression pattern was assigned ‘up’- or ‘down’-regulated status based on the sign of its correlation. Here, 290 SD genes were upregulated and 370 were down-regulated.

### Pathways Altered in Sleep Deprived Subjects

To identify pathways altered in sleep deprivation, we took the two largest cohorts of significant genes, the sustained and transient patterns, and separated them by direction of change. Each subgroup was uploaded to the DAVID website (Methods) and subjected to overrepresentation analysis. The delayed and linear patterns contained significantly more genes than expected by chance, but had too few genes for DAVID analysis. Therefore results from these templates were considered individually (Table 1). A sustained increase glucocorticoid stimulus, a transient decrease in cell morphogenesis/neurogenesis, and a sustained decrease in neuronal/synaptic signaling were identified. These results are largely consistent with prior work on SD and interpretations are presented in Discussion.

**Table 1:** Pathways influenced by sleep deprivation.

Sustained	
Increased	Decreased
51384: **response to glucocorticoid stimulus** (# 8, p = 0.0083) *Bad, Ghr,* *Hmgb1, Il6r, Plat, Ptgs2, Sgk, Sult1a1*	45202: **synapse** (# 23, p = 0.016) *Add1, Chrna7, Cplx1, Dlg2, Erbb3, Gabra3, Gad2, Gpr51, Grm7, Htr3a, Myh9, P2rx4, Prkar1a, Rabac1, Rgs19ip1, Sdfr1, Slc2a3, Snca, Sparcl1, Syngr1, Synpo, Syt3, Unc13h1*
32355: **response to estradiol stimulus** (#6, p = 0.023) *Bad, Cryab, Gpx1,* *Insig1, Ptgs2, Ptk2*	06538: **glutamate catabolic process** (# 3, p = 0.029) *Gad2, Glud1, Got2*
00165: **MAPKKK cascade** (# 7, p = 0.024) *Cryab, Dusp6, Ghr, Grm4, Mapk14,* *Ptk2, Raf1*	12506: **vesicle membrane** (# 9, p = 0.04) *Ap2b1, Camk2g, Cftr, Gad2, Rgs19ip1, Slc2a3, Snca, Syngr1, Tmp21*
30198: **extracellular matrix organization** (#5, p = 0.037*) Apex1, Col11a1,* *Fn16974, Gadd45a, Hmgb1, Hmgb2, Mapk14, Msh2, P4ha1, Ptk2, Serpinh1, Sgk*	
43565: **sequence-specific DNA binding** (#9, p = 0.039) *Cebpb, Cutl1,* *Hnrpab, Msh2, Neurod1, Nsep1, Pou3f1, Sox10, Znf148*	
48869: **cellular developmental process** (#29, p = 0.04) *Actb, C1s, Cap1,* *Cebpb, Celsr3, Clcn2, Col11a1, Cr16, Cutl1, Dusp6, Fn1, Gpx1, Grm4, Hes1, Hmgb1,* *Hmgb2, Hnrpab, Homer1, Ifrd1, Mapk14, Msh2, Myt1l, Neurod1, Pou3f1, Ptk2, Rbp4,* *Sgk, Slc1a3, Sox*	
00902: **cell morphogenesis** (#11, p = 0.047) *Actb, Cap1, Celsr3, Fn1, Hes1, Hmgb1, Hnrpab, Mapk14, Ptk2, Sgk, Slc1a3*	
44260: **cellular macromolecule metabolic process** (#43, p = 0.048*)* *Apex1, Ccnl, Cebpb, Col11a1, Cryab, Cutl1, Dusp6, Fn1, Galnt1, Ghr, Gpx1, Grm4, Hes1,* *Hmgb1, Hmgb2, hnRNPA3, Hnrpa1, Hnrpu, Klf9, Mapk14, Mrpl23, Msh2, Myt1l, Neurod1,* *Nfia, Nsep1, P4ha1, Per2, Pou3f1, Ppig, Prkcl1, Psma2, PSMD1, Ptk2, Raf1, Rod1, Rpl10,* *Safb, Sgk, Sox10, Top1, Znf148, Znf265*	
**Transient**	
50790: **regulation of catalytic activity** (#21, p = 0.0058) *Akt2, Arg2, Atpi,* *Cd24, Cycs, Eif2ak3, F3, Fkbp1a, Frap1, Grm1, Hsp60, Hspa5, Mapk8ip, Plcd1,* *Psma7, Psmd4, Pthlh, Rb1, Rbl2, Ssbp1, Vldlr*	22803: **passive transmembrane transporter activity** (# 12, p = 0.014) *Cacna1g, Cacnb3, Chrnb4, Gjb1, Grik5, Grin2d, Kcnj14, LOC293497, Mip, Scn1b, Snap25, Z49858*
43255: **regulation of carbohydrate biosynthetic process** (#4, p = 0.013)*Akt2, Frap1, Plcd1, Ppp1cb*	06970: **response to osmotic stress** (# 5, p = 0.018*) Avp, Avpr1, Bax, Kmo, Sord*
04435: **phosphoinositide phospholipase C activity** (#3, p = 0.025)*Plcb1, Plcd1, LOC84587*	42175: **nuclear envelope-endoplasmic reticulum network** (#8, p = 0.018) *Bax, Cyp2e1, Fdft1, Gjb1, Oprs1, Pemt, Psen1, Sqle*
06919**: activation of caspase activity** (#4, p = 0.027) *Cycs, F3,* *Eif2ak3, Hsp60*	44431: **Golgi apparatus part** (#10, p = 0.035) *Cspg5, Cyp2e1, Loc192276, LOC246768, Pcsk7, Siat1, Siat8b, Snap25, Sod3, Vamp2*
05509: **calcium ion binding** (#15, p = 0.037) *Cdh8, Chga, Grp94, Itpr1,* *LOC84587, Lre3, P5, Plcb1, Plcd1, Pthlh, Pva, Ret, Tpt1, Trrp1, Vldlr*	48667: **cell morphogenesis involved in neuron differentiation** (#9, p = 0.041) *Cdk5r, Cnp1, Cspg5, Ephb1, Fez1, Ncaml1, Snap25, Spr, Unc5h2*
10468**: regulation of gene expression** (#24, p = 0.038) *Akt2, Cdc5l, Cited2,* *Csda, Dbp, Eif2ak3, Eif4g2, Frap1, Gtf2a2, Hivep1, Hivep2, Hsf1, Hsp60, Litaf,* *LOC81816, Mapk8ip, Nr1d2, Pax6, Pou3f3, Pthlh, Rb1, Rbl2, Roaz, Znf146*	
19222: **regulation of metabolic process** (#36, p = 0.045) *Akt2, Arg2, Atpi,* *Cd24, Cdc5l, Cited2, Csda, Dbp, Eif2ak3, Eif4g2, Epac, Fkbp1a, Frap1, Grm1, Gtf2a2,* *Hivep1, Hivep2, Hsf1, Hsp60, Litaf, LOC81816, Mapk8ip, Nr1d2, Pax6, Pdgfra, Plcd1,* *Pou3f3, Ppp1cb, Psma7, Psmd4, Pthlh, Rb1, Rbl2, Roaz, Vldlr, Znf146*	
34645**: cellular macromolecule biosynthetic process** (#22, p = 0.05)*Cdc5l, Csda, Dbp, Eif2ak3, Eif5, Gtf2a2, Gyg, Hivep2, Litaf, Nr1d2, Pax6, Rb1, Rbl2, Roaz,* *Rpl14, Rpl17, Rpl21, Rpl32, Rps15a, Slipr, Ssbp1, Znf146*	

Genes with sustained or transient expression patterns (Fig. 2A, B) were separated by direction and analyzed by functional grouping overrepresentation analysis (Methods). Representative categories for each pattern and direction are listed with Gene Ontology ID and description. The number of significant genes (**#**) and DAVID modified Fisher exact score p-value are given in parentheses, followed by alphabetical listing of gene symbols within each pathway.

### Contrasting Sleep Deprivation and Stress Effects

**Figure 4:**
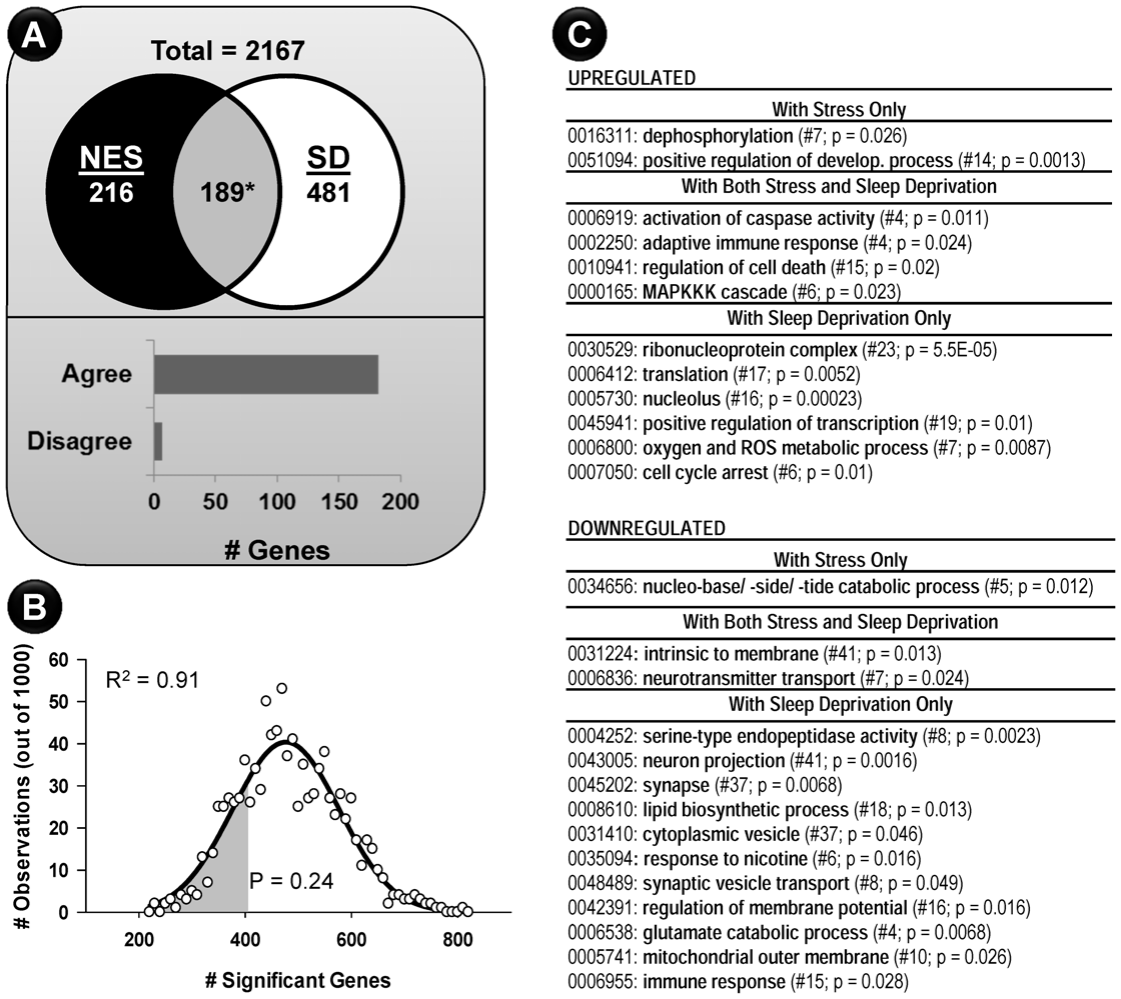
Novel environment stress (NES) elicited a transcriptional response highly similar to that seen with SD. A separate cohort of animals exposed to NES were subjected to hippocampal microarray analysis. Of the 2167 filtered genes, 405 were identified as significant in the stress study (p≤0.05; 1-way ANOVA; FDR  = 0.18). **A.** Venn diagram comparing stress (black circle) and SD (SD- white circle) array results reveals a highly significant overlap of 189 genes significant in both studies (* p  = 1.55×10^−8^; binomial test). Directional analysis revealed that 96% (182/189; 81 upregulated; 101 downregulated) of these overlapping genes agreed in direction of change. **B.** NES was powered by 21 microarrays, while SD was powered by 53. Because of this discrepancy, the greater number of genes found in SD could reflect increased discovery power, rather than a stronger effect of SD. To test this, we iteratively selected subsets of 21 arrays from the 53 used in the SD study and tested for significance. This was repeated 1000 times and in each iteration, the number of genes significant (p≤0.05, 1-way ANOVA) were counted. The results from all 1000 iterations are plotted as a frequency histogram (open circles). This was well-fit by a Gaussian function (heavy black line, p<0.0001, R^2^ = 0.91) with a peak of 476.4- meaning that, on average if only 21 chips had been used in the SD study, we would predict that 476 genes would be found significant. Using the fit function, and the observation that 405 genes were found in the NES study, we fail to support the hypothesis that SD finds more genes than NES (p  = 0.24; integrated area under the curve- gray). **C.** Gene Ontology Analysis for genes in each region/direction of change within the Venn diagram.

The SD protocol used here has been shown to elicit a stress response [27]. To help define sleep vs. stress-related changes, we performed a third experiment in which subjects were exposed to novel environment stress (NES) for 24 or 72 hours. This study was done in parallel with, and shares the same home cage (HC) controls as, cohort 1 of the SD study. The microarray analysis process was highly similar to that used for SD analysis (not shown). Significant results (405 genes, p≤0.05, FDR  = 0.18, 1-way ANOVA) were contrasted with results from the SD analysis (Fig. 4A). The number of genes commonly regulated by the two experimental conditions was highly unlikely to have occurred by chance.

NES elicited a smaller transcriptional response than SD. However, the NES analysis had fewer arrays (N  = 21) than SD (N  = 53), likely resulting in reduced statistical discovery power. To determine whether this difference in N could explain the reduction in number of significant genes, we performed a resampling analysis in which the SD study was artificially restricted to only 21 observations. Among the 53 SD chips, a subset of 21 was tested, and the number of significant genes (1-way ANOVA; p≤0.05) noted. This process was repeated 1000 times with different randomly selected subsets of 21 chips, generating 1000 estimates of the number of significant genes expected if the SD study had only 21 chips. These estimates were plotted as a frequency histogram (Fig. 4B) and were well fit by a Gaussian distribution. The peak of this distribution (∼476) represents the average number of significant genes we predict would be significant if N  = 21 in the SD study. Superimposing the number of genes actually found significant in the NES study (405 genes- gray area, Fig. 4B) revealed that the ‘transcriptional magnitude of effect’, between NES and SD was not significantly different. That is, although 405 NES genes (observed) is less than 476 (predicted) SD genes, it is not significantly less. Therefore, the high degree of overlap among genes changed in NES and SD, the agreement in direction of that change, and, when compensating for statistical power differences, the relative similarity in the transcriptional strength of these experimental manipulations all suggest that the functional processes associated with SD and NES should be highly similar.

To test this, each region of the Venn diagram (Fig. 4A) was separated by direction of change and analyzed by functional overrepresentation. We tested the prediction that the two treatments (SD and NES) would show relatively similar functional grouping results and that, if anything, SD would show a more refined dissection of functional groups identified in both SD and NES due to their similarity at the overlap level. Surprisingly, only the upregulated immune and apoptosis pathways supported this hypothesis. SD showed significant and opposite influences on a separate cohort of immune-related genes, and exclusive upregulation of machinery involved in mRNA translation to protein, and downregulation of neuronal components (illustrated in Fig. 4C).

### Comparison to other SD Studies

The overlap analysis approach used for the following comparisons has a strong tendency to increase confidence in true positive findings at the expense of false negatives [40]. It is also important to note that differences across laboratories, SD techniques, brain regions studied and species used likely account for disagreements among studies. Thus, genes identified in multiple studies should represent robust SD responses that are less likely to be species and/or brain region-dependent.

Complete microarray datasets are available for two mouse brain studies of sleep deprivation: GSE 6514 [53]; GSE 23628 [54]. Downloaded and compared to our data set, 495 total genes were present in all three studies. Significant genes: our study  = 169, Mackiewicz  = 148, and Thompson  = 63. By chance, 1 gene should be found significantly changed in the same direction by all three studies, while 4 were observed to change (p  = 0.024, binomial test). Downregulated in all three studies: Cirbp (cold-inducible binding protein), S100a1 (S100 calcium binding protein A1) and Ednrb (endothelin receptor type 1). Interestingly, the three downregulated genes were also robustly downregulated in our NES group.

Three studies examined SD-related transcriptional changes in rat hippocampus [50], rat cerebral cortex [55], or mouse forebrain [56]. However, lists of selected significant genes, rather than complete data sets, are available for these studies. Thus, we restricted reports to genes found within our filtered data set, and used a statistical approach similar to that employed in DAVID analysis (679/2167 = 35% of our data set significant; selected genes then, 17.5% chance of significance and directional agreement across studies). Conti et al report 15 significant genes, 7 of which were significant and changed in the same direction in our study. Mongrain et al report 141 significant results (exclusively among control vs. sleep deprived intact animals). Fifty of these are significant and agree in direction in the present work. Cirelli et al report 28 genes, 12 of which were significant in our study. All of these comparisons were significant (p≤0.05; binomial test) and Table 1 is annotated with these findings. Interestingly, Homer1 was significantly upregulated in all five prior microarray sleep deprivation studies, as well as in our own study, and was not significantly influenced by NES.

### Comparison to Hippocampal Aging

**Figure 5:**
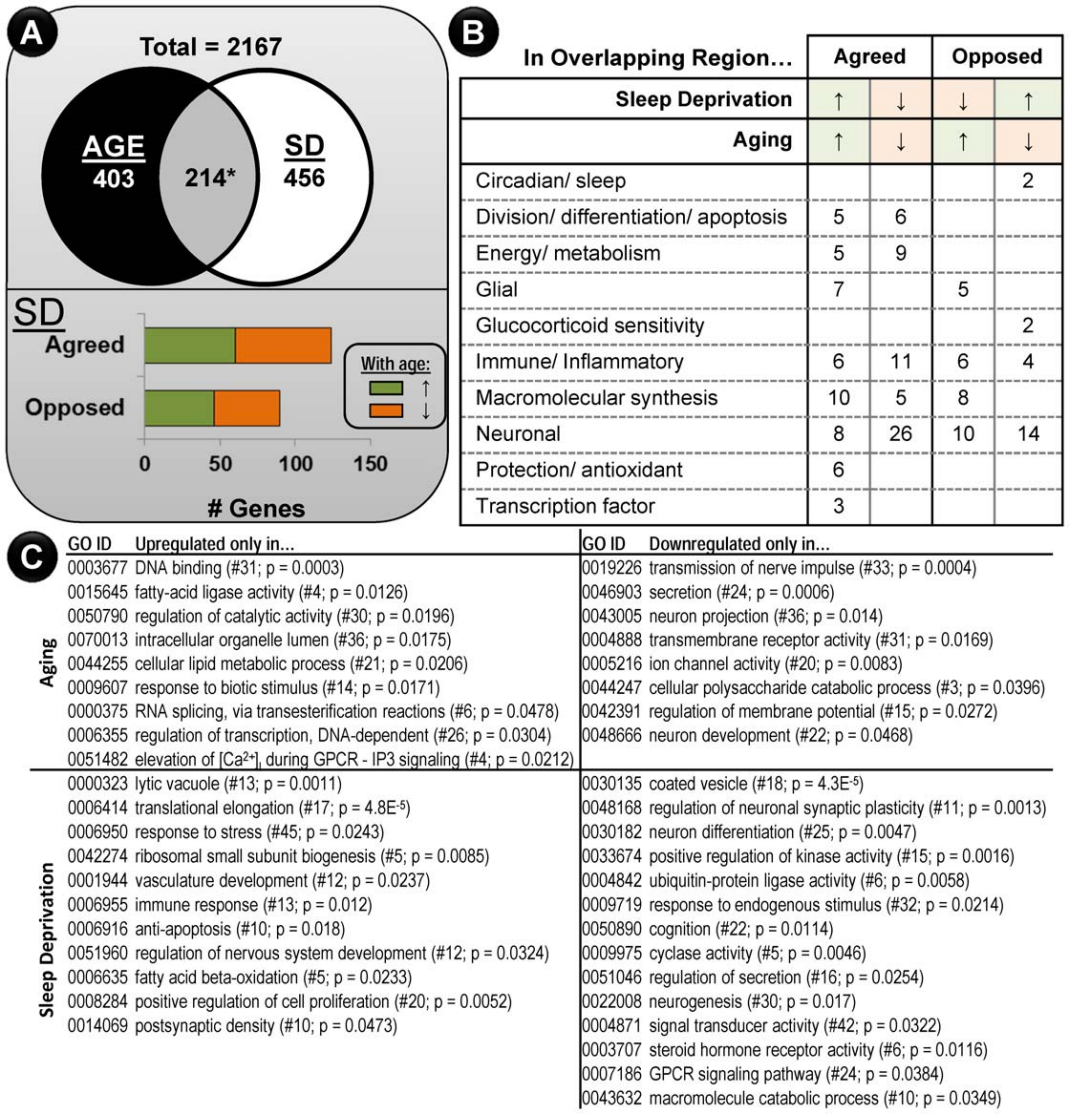
SD and aging influence a similar set of genes. A. Unlike NES (Fig. 4), the significant overlap between aging and SD (* p  = 0.028, binomial test) contained many genes whose change with age was opposite to that in SD. **B.** 158/214 genes in the overlap were manually assigned to one of 10 heuristic categories. Because of this approach, no statistical overrepresentation p-values are possible. The number of genes from within each quadrant of the overlap (agreed with SD, aging up, aging down; disagreed in SD- aging up, aging down) are shown. Genes in each category are listed in Results. **C.** Functional categorization genes regulated exclusively by Aging (upper) or Sleep Deprivation (lower) are separated based on direction of change (Left: Upregulated; Right: Downregulated).

We tested the prediction that SD would cause an aging-like shift in hippocampal gene expression by assembling a composite set of aging genes in rat hippocampus based on three studies [31], [32], [33]. The simplified ‘aging union’ results (up- or downregulated in at least one of the prior aging studies- see Methods) are annotated in Table S1. 617 genes changed with aging and 670 changed with SD. Functional overrepresentation analysis (see Methods) was possible (Fig. 5C) for genes uniquely regulated by SD (456 genes) or Aging (403 genes). Aging showed a selective upregulation of pathways related to transcription, lipid metabolism, and Ca^2+^ signaling. Further, downregulated categories were almost exclusively related to neuronal function. Interestingly, sleep deprivation appeared to also downregulate a strong, but different, cohort of neuronal genes (Fig. 5C lower right), suggesting that SD and aging may have additive influences on brain function. Upregulated exclusive SD genes included processes related to protein handling and degradation, as well as well-characterized upregulation of stress hormone stimulus pathways.

A significant subset (214 genes, p  = 0.028, binomial test; Fig. 5A) are changed by both SD and aging. Therefore, as with the comparison between SD and NES (Fig. 4), aging and SD appear to exert influence over a common set of genes. However, unlike the very strong tendency for SD and NES gene expression to agree in direction (Fig. 4A), this could not be said of aging and SD (Fig. 5A, lower), where significant genes were split- 128/214 agreed in direction. We partitioned the overlapping 214 genes into four quadrants based on direction of change in aging and SD. Because the resulting profiles were too small for reliable DAVID functional grouping statistical analysis, genes were manually categorized (Fig. 5B- and see below). Immune/inflammatory, neuronal, and macromolecular synthesis categories showed the largest effects. Division/differentiation/apoptosis, energy metabolism, protection/antioxidant, and transcription factor changes appeared similar in aging and SD. Glial changes were upregulated in aging, and a subset of those upregulated changes moved in the opposite direction with SD. Finally, glucocorticoid sensitivity and circadian/sleep were both downregulated with age and upregulated by SD. Individual genes listed in each condition or category, along with notes regarding putative function, are included below.

#### 1) Upregulated in both aging and SD (60 genes)

Division/differentiation/apoptosis: *Ap1b1, Cd24, Cdc5l, Msh2, Vcp*

Energy/metabolism: *Aldoc, Ech1, Gatm, Sds, Slc2a1*

Glial: *Adk, Cryab, Ocln, Mt1a, Oplah, Pmp22* (although exclusively expressed in peripheral nervous system, mRNA is detected in CNS, see Allen Brain Atlas- [57]), *Slc1a3*

Immune/inflammatory: C1qb, Cyp4f4, Fn1*, Hmgb2, Ndrg2, Ptgs1* (* inflammatory)

Macromolecular synthesis: Csda*, Eif4ebp1*, Eif5, Rpl10, Rpl21, Rpl23, Rps15, Rps21, Rps5, Rps7 (* suppresses synthesis)

Neuronal: *Adra1d, Fxyd1, Gng5, Grip2, Ptpn1, Ralgds, Slc1a3, Sult1a1*

Protection/antioxidant: Cited2, Gpx1, Prdx6, Sepp1, Sod3, *Xdh (*pro-oxidation)

Transcription factor: *Dbp, Neurod1, Nfia*

#### 2) Downregulated in both aging and SD (64 genes)

Division/differentiation/apoptosis: *Egr1, Igf2, Lxn, Met, Ngfrap1, Sc65*

Energy/metabolism: *Acat1, Atp9a, Amacr, Atp1a3, Dnm1l, Fez1, Got2, Ivd, Tst*

Immune/inflammatory: *Add1, Ap3m2, Csrp2, Cx3cl1, Dcn, Dpp3, Ide, Lamc1, Lrpap1, Muc1, Tubb5 (primarily fibril formation and inflammation-related genes)*

Macromolecular synthesis: Cyp51, Faah, Fapb3, Hmgcr*, Sqle* (* rate-limiting enzymes in the sterol biosynthesis pathway)

Neuronal: *Accn1, Adcy5, Agrn, Ap2b1, App, Calb1, Calb2, Capns1, Crmp1, Fez1, Gabbr1, Gda, Htr2c, Kcns3, Nnat, Pctk1, Pkia, Por, Ppp2r2c, Prkar1a, Ptpra, Ptprn2, Rab12, Slc1a1, Snca, Syt4*

#### 3) Upregulated in aging and downregulated by SD (46 genes)

Glial: *Fgfr2, Gfap, Gjb1, Rab13, Tip2*

Immune/inflammatory: Dbnl, Grn*, Lamp1*, Litaf*, Plat*, Psme1 (* inflammatory)

Macromolecular synthesis: *Gpam, Insig1, Magat1, Pgr, Phgdh, Ppap2c, Rpl29, Srebf1*

Neuronal: *Akr7a2, Bin1, Cpd, Cplx2, Klk6, Nln, Pgr, Pla2g2c, Syngr1, Vamp1*

#### 4) Downregulated in aging and upregulated by SD (44 genes)

Neuronal: *Apbb3, Begain, Calr, Celsr3, Cgref1, Chga, Hspa4, Ifrd1, Marcks, Mark3, Npy, Rgs14, Tac1, Vgf* (Note: *Tac1, Ifrd1,* and *Npy* may also be related to immune or inflammatory signaling)

Immune/inflammatory: *Arg2, Col11a1, Serpinh1, Tpm1* (generally opposes other inflammatory signals)

Glucocorticoid sensitivity: *Chrbp1, Fkbp4* (expression opposes glucocorticoid action)

Circadian/sleep: *Homer1, Per2*

## Discussion

We used a modified ‘flower pot’ method to provide sustained Sleep Deprivation (SD) [28], [58] and included a novel environment stress (NES) control in order to separate the effects of SD from those of NES on hippocampal gene expression. Transcriptional profiling of the hippocampal CA1 brain region was selected because the hippocampus is well-understood to be negatively affected by SD, aging, and stress. We also tested whether SD would elicit aging-like changes in the transcriptional profile. Significant overlapping gene expression profiles were observed for SD and stress, but much less so for SD and aging. Our studies of the transcriptional effects of SD also identified candidate druggable targets for the development of new sleep countermeasures. In addition to supporting a role for certain genes in sleep modulation (e.g., Homer1), our studies identified some novel pathways as potential targets for intervention in SD, most notably those involved in synaptic function.

**SD has stress-like components.** In agreement with prior studies, body weight loss and corticosterone elevation (Fig. 1A,C) suggest that animals perceived the SD environment as a stressor [59]. Animals exposed to novel environment stress (NES) had a strong directional agreement with SD (Fig. 4) for ∼1/3 of all significant SD genes. Based on that overlap, and on the relatively smaller effect of NES, we hypothesized that NES subjects experienced a similar but lower intensity combination of stress and sleep loss compared to SD subjects. To test this, we controlled for the lower statistical power of the NES study (Fig. 4B), and found that the transcriptional magnitude of effect was not significantly different in SD and NES. Therefore, it appears that the NES animals manifested a transcriptional response similar to that of SD. However, selective SD pathways not found in NES (energy metabolism, macromolecular synthesis, and neuronal function) support some of the most prominent mechanistic hypotheses of sleep function in brain tissue [20], [60]. Thus, SD appeared to exert a composite effect [30], [61] on the hippocampal transcriptome consistent with both stress and sleep’s hypothesized function(s). Elegant work depriving certain sleep stages without enriching wake [62], or invoking SD and its concomitant stressors in the absence of stress-related hormone secretion [56] likely will help to elucidate the separate contributions of these phenomena.

### Protein Measures/Validation

We also tested the hypothesis that mRNA level changes identified by microarray translate to protein using a separate cohort of similarly sleep-deprived animals. Western blots for Adrb2, Agrin, Gria2, and Neurexin support this. Although Sgk1 and Slc1a1 failed to reach significance in the protein measures, they showed the same direction of change and it may be unreasonable to expect complete agreement using overlap comparisons. The overall finding of 4 genes agreeing, and two more agreeing in direction of change, particularly in a separate set of subjects measuring a different type of molecule, appears to constitute reasonable corroboration. Additionally, prior microarray SD studies in brain tissue [50], [54], [56], [60] also validate these findings (noted in Table S1; see [63] for discussion on limitations of this approach).

### Relationship to Aging

Prior studies have shown that sleep depriving young subjects can result in aging-like behavioral and metabolic changes [62]. Further, in humans and animal models, sleep loss appears to be a normal consequence of the aging process, and could be a proximate cause of age-related functional deficits [64]. We predicted that artificially restricting sleep in young animals would evoke an aging-like transcriptional profile. We constructed a unified aging profile based on three different rat hippocampal studies. At an initial level, our hypothesis was supported as we found a highly significant subset of genes influenced by both SD and aging (Fig. 5). Surprisingly, many genes significant in both SD and aging changed in opposite directions, suggesting a more complex relationship.

**Figure 6:**
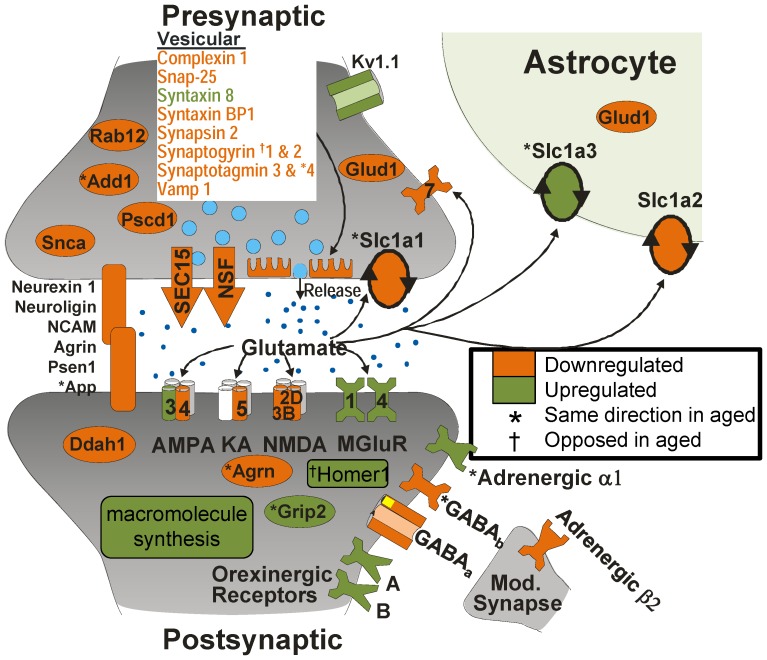
SD targets molecules associated with the glutamatergic synapse. We developed an *a priori* defined list of genes (101) reported to play a role in glutamatergic neurotransmission. 46 were significantly altered with SD (35 decreasing, 15 increasing. A high proportion of downregulated messages were associated with presynaptic neurotransmitter release and cell adhesion. As a process, macromolecular synthesis appears increased. Genes also found to change with age are noted with an (*- agreed; † opposed). Abbreviations: *Add1*- adducin 1 α; *Agrn*- agrin; *Ddah1*- dimethyl arginine dimethyl aminohydrolase; *Glud1*- glutamate dehydrogenase 1; Glutamate transporters (*Slc1a1*- excitatory amino acid transporter 3; *Grip2*- glutamate receptor interacting protein 2; *Slc1a2*- excitatory amino acid transporter 2, *Slc1a3*- excitatory amino acid transporter 1); *Kv1.1*- shaker K+ channel; *Nsf*- n-ethylamide sensitive factor; *Psen1*- presenilin 1; *Pscd1*- pleckstrin homology; *Sec15*- secretory factor 15; *Snca*- alpha synuclein.

Overall, a significantly greater number of genes than expected by chance were regulated by both aging and sleep deprivation (Fig. 5). However, contrary to our hypothesis, genes within this ‘aging and SD transcriptional window’ were not generally moved in the same direction by the two phenomena. Although our prediction’s first component (i.e., that more genes than expected by chance would be influenced) was supported, the second component (that those overlapping genes would be similarly influenced by the two phenomena), was not. This suggests that age-related changes interact in a more complex manner with SD-related genes. A build-up of Homer1 expression with increasing sleep deprivation seen here has been reported in other work [50], [54], [55], [56], [65], [66], [67]. Conversely, multiple studies have shown a decrease in Homer1 expression, with aging [31], [32], [33], [68] suggesting that Homer1, and possibly other candidate sleep-pressure signaling systems, may serve as a lynch pin for discrepancies between young and aged sleep behavior and molecular profiles. Interestingly, with the exception of Homer 1 and Synaptogyrin 1, analysis focused on synapse-related gene expression (Fig. 6) points to aging’s similarity to SD’s influence (moves Add1, Agrin, App, Grip2, Slc1a1, Slc1a3, Synaptogagmin 4, GABA_B_, and the Adrenergic α1 receptor in the same direction as SD). Future studies examining the influence of stress, stress hormone, and their interaction with sleep and Homer1 expression with age may help to further clarify these issues.

Overlapping genes were categorized by direction of change in aging and SD, as well as by putative function (Fig. 5B). Processes that changed with aging and were apparently recapitulated with SD included cell differentiation/apoptosis, energy, antioxidant and transcription factor activity. Among genes that disagreed between aging and SD, two that influence sensitivity to glucocorticoid, Chrbp and Fkbp4 (also upregulated in a prior SD study- [56]), were upregulated in SD and downregulated with age in the present analysis. Changes in the expression of these candidate molecules may dampen glucocorticoid’s influence on immune/inflammatory and glial activity with age [25]. Intriguing parallels to work on other steroid hormones [69], suggest that, with age, the brain may shift its response to glucocorticoids. Whether such mechanisms may involve nuclear or non-nuclear receptor pathways [70] remains to be determined.

Two sleep-related genes, Per2 and Homer1, were suppressed with age but upregulated with SD. Per2 is a circadian clock gene upregulated in prior SD studies [56] as well as in our NES treatment group, suggesting it may not be a purely SD-related finding. Homer1 was among the few genes that showed a linear increase with extended SD and was not influenced by NES. Further, multiple SD studies have also reported Homer1 upregulation with SD [50], [54], [55], [56], [66]. Homer1 may play an important role in sleep pressure signaling [65], [66], [67], [71]. Because the brain exhibits less deep-sleep with age in both humans [64], [72] and animal models [73], [74], [75], [76], [77], [78], we speculate that Homer1’s consistent downregulation with aging [31], [32], [33] could constitute a broken molecular switch leading to a loss of deep sleep with age.

### Therapeutic Targets for Intervention in SD

Results point to a focused effect on mRNA associated with synaptic function. We constructed an idealized hippocampal glutamatergic synapse (Fig. 6), and superimposed SD profile results (the calcium-sensing protein S100A1 is included because it co-localizes with synapsin in brain tissue [79]). Gene products were identified using literature and ontology database searches: 46 were significantly altered by sleep deprivation- the majority downregulated. Results suggest SD-induced synaptic efficacy and macromolecular synthesis changes, consistent with previous work [22], [60].

In keeping with the proposed mechanisms of action for current SD countermeasures (e.g., caffeine, amphetamine, modafinil, the ampakine CX717), there appears to be a deficit in glutamatergic signaling with SD. Interestingly, *Chga* (chromogranin A) upregulation has been reported to suppress presynaptic vesicular release components [80], [81] and may, at least in part, play an upstream role in mRNA expression changes associated with the pre-synapse. This may help to explain how SD-countering drugs [82] can exert their effects and highlights the potential clinical importance of astrocytic [83], orexinergic and adrenergic systems. Results also suggest that drugs facilitating Ca^2+^-dependent vesicular release (one of caffeine’s proposed mechanisms of action), neurotransmitter re-uptake block (one of amphetamine’s proposed mechanisms of action) may counter SD’s effects. Among newer agents, the ampakine CX717 is proposed to exert its wake-promoting effects via enhanced glutamatergic signaling [82], [84]. Conversely, drugs that constrain neuronal activity via: reduced sustained high frequency repetitive discharge (e.g, phenytoin); enhanced inhibitory surround (e.g., phenobarbital); or disrupted vesicular release (e.g., levetiracetam) facilitate sleep [85]. The development of drugs that enhance presynaptic release, a mechanism of action relatively unexplored by current wake-promoting agents, seems a valid target for the development of future sleep countermeasures.

No currently available sleep countermeasures allow human ‘normal wake’ performance over extended (three or more days) periods of time. Frank limitations include dissociative/psychotic behavior after prolonged exposure, risk of developing tolerance/dependence on the agents being used, and lowered seizure thresholds [86]. Our microarray results indicate that synaptic function-related mRNA expression changes do not occur in a vacuum. Among other categories of change, the reduction in intercellular adhesion molecule expression may be of particular importance. These molecules are pleiotropic and in addition to playing a role in inflammation and immune signaling, also help maintain synaptic juxtaposition. We speculate that their downregulation in SD could leading to aberrant synaptic signaling. Thus, the development of drugs targeting the stabilization of intercellular adhesion molecules may help to extend the duration of action for stimulant-based sleep countering agents.1.

#### Summary

Our findings support long-standing observations that SD elicits physiologic and transcriptional responses with stress-like features. Neuronal synaptic gene expression changes may help to explain the benefits and limitations of current stimulant-based SD-countering therapeutics and points to novel targets in presynaptic vesicular release and neural cell adhesion for development of future SD-countering drugs. Significantly more genes than expected by chance were commonly regulated by both aging and SD. Although many were driven in the same direction by these two phenomena, we could not fully support the hypothesis that SD evokes an aging-like transcriptional change. Genes showing significant but opposite expression changes in SD and aging, including *Chrbp*, *Fkbp4*, *Homer1* and *Per2*, are candidates for further study of the interplay between SD and aging. Compared to prior studies showing increased inflammatory signaling with stress, our novel environment stress (NES) protocol did not influence inflammatory gene expression to an appreciable extent, and SD suppressed those signals. This suggests that SD in our hands was either a weak stressor, or that it has an effect mechanistically distinct from that of stress on immune signaling.

## Supporting Information

Table S1 **Genes whose expression levels were significantly changed as a consequence of sleep deprivation are listed.** Probe set IDs (Affymetrix), annotated gene symbols (hyperlinked to Uniprot), and gene titles are shown. Subsequent columns contain comparisons to other array studies as noted in Methods and Results (blank if no results were found). Both significance, as well as template (see Fig. 2) with which results best fit are provided for novel environment stress (NES), as well as sleep deprivation (SD). For SD, means ± SEM for each treatment group are also provided.
(XLSX)
